# Mesenchymal and stemness circulating tumor cells in early breast cancer diagnosis

**DOI:** 10.1186/1471-2407-12-114

**Published:** 2012-03-23

**Authors:** Guislaine Barrière, Alain Riouallon, Joël Renaudie, Michel Tartary, Michel Rigaud

**Affiliations:** 1 Astralab clinical laboratory, 7-11 Avenue de Lattre de Tassigny, 87000 Limoges, France; 2 Department of gynecology and surgery, Clinique du Colombier, 92 avenue Albert Thomas, 87100 Limoges, France

## Abstract

**Background:**

Epithelial mesenchymal transition (EMT) is a crucial event likely involved in dissemination of epithelial cancer cells. This process enables them to acquire migratory/invasive properties, contributing to tumor and metastatic spread. To know if this event is an early one in breast cancer, we developed a clinical trial. The aim of this protocol was to detect circulating tumor cells endowed with mesenchymal and/or stemness characteristics, at the time of initial diagnosis. Breast cancer patients (n = 61), without visceral or bone metastasis were enrolled and analysis of these dedifferentiated circulating tumor cells (ddCTC) was realized.

**Methods:**

*AdnaGen *method was used for enrichment cell selection. Then, ddCTC were characterized by RT-PCR study of the following genes: PI3Kα, Akt-2, Twist1 (EMT markers) and ALDH1, Bmi1 and CD44 (stemness indicators).

**Results:**

Among the studied primary breast cancer cohort, presence of ddCTC was detected in 39% of cases. This positivity is independant from tumor clinicopathological factors apart from the lymph node status.

**Conclusions:**

Our data uniquely demonstrated that *in vivo *EMT occurs in the primary tumors and is associated with an enhanced ability of tumor cells to intravasate in the early phase of cancer disease. These results suggest that analysis of circulating tumor cells focused on cells showing mesenchymal or stemness characteristics might facilitate assessment of new drugs in clinical trials.

## Background

Many trials are currently focusing on circulating tumor cells (CTC) in peripheral blood and publications have described association of CTC with worse prognosis [1]. The term CTC encompasses all types of cells which are considered as foreign entities in the blood having some cancerous characters. Often, in this whole population, cells with tumorigenic potential are not detected. Results are essentially supported by cell counting data arising from CellSearch (Veridex, Raritan, NJ). This method has been cleared by FDA but is not endorsed, like others, by ASCO recommendations. Recent studies have suggested that CellSearch technique may underestimate the number of CTC [2]. Blood intravasation of cancer cells from a primary breast carcinoma is an early event, leading to metastasis [3,4]. Klein *et al *proposed a parallel progression between early primary tumor and dissemination of tumor cells [5]. Evidence has emerged that CTC present a heterogeneity as the one described for primary tumors. Subpopulations of CTC: cancer stem cells, tumor amplifying cells (*i.e *progenitors) and tumor initiating cells arise from epithelial cancer cells of the primary tumor undergoing epithelial mesenchymal transition (EMT) program [6,7]. Targeting these types of cells, cancer stem cells [8] and mesenchymal cells in the blood, would provide more clinically relevant prognosis and predictive information than simple CTC counting. Their analysis should help in a more tight follow-up of the disease progression.

For epithelial malignancies, EMT is a crucial event in the dissemination of cancer cells. Carcinoma epithelial cells are linked to their neighbours and to basal membranes by protein junctions which are abolished when cells acquire a migratory mesenchymal phenotype. The EMT process is regulated by pleiotropic cytokines such as TGFβ acting through the "cadherin switch" [9]. Cell adhesion proteins are transcriptionally repressed by classical EMT-related signaling pathways. Among them, PI3Kα/Akt-2/mTOR network acts to modulate cell survival, migration and resistance to anoikis and apoptosis [10,11]. Twist transcriptionally up-regulates Akt-2 in breast cancer cells leading to increase migration and invasion [12]. EMT is associated with acquisition of stem cell-like characteristics: Bmi1, a protein that promotes and maintains self-renewal, is a direct transcriptional target of the EMT inducer Twist1 [13]. Studies indicated that ALDH1, responsible for oxidation of retinol to retinoic acid, is a potent marker of breast cancer stem cells [14]. In breast cancer, CD44^+^/CD24^-/low ^cells are known to be highly tumorigenic in immunodeficient mice [15]. These markers are useful for identification of CTC having EMT and/or stemness phenotypes. We named dedifferentiated circulating tumor cells (ddCTC) only the subpopulations having one or several mesenchymal and/or stemness characteristics. Due to these considerations, we started a preliminary study for detection of ddCTC at the time of the early breast cancer diagnosis. These analyses were performed before any therapy. EMT and stemness marker expressions were analyzed in order to identify ddCTC.

## Methods

### Patient population

The study was conducted at the department of gynecological surgery (Private Hospital Clinique le Colombier) and Astralab laboratory (Department of specialized medical analyses) in Limoges Fr. Since January 2011, patients have been included and blood samples collected before any therapy. Among them, 61 patients were selected provided they showed the following criteria: age 40-75 years, breast cancer diagnosis confirmed by pathologist analysis of the primary tumor, absence of bone, visceral, cerebral metastasis (controlateral breast mammography, liver ultrasonography and entire body bone scanning). Axillary lymph node invasion was assessed.

This protocol was performed with the approval of the appropriate ethic local committee (Comité de protection des personnes Sud-Ouest et Outre-mer IV. France) and was in compliance with the Helsinki Declaration.

### Sampling of biological material

Blood samples of 7 ml were collected for cellular enrichment with *AdnaCollect *tubes (AdnaGen AG, Langenhagen, Germany) and were stored and shipped in the dark at 4-8°C. Samples were analysed within 24 hours. Blood collection tubes contain EDTA and an agent preventing illegitimate RNA expression. Selection and detection steps of CTC were led as published [16,17]. They are briefly summarized.

### Selection of CTC

Tumor cells enrichment was realized by using immunomagnetic beads coated with three antibodies, one against EpCam, two others against Muc-1. One of the latter directed to an underglycosylated epitope, stably not accessible in other blood cells, is more specific of cancer cells. During this enrichment step of mesenchymal and stem breast cancer cells a special washing buffer procedure allowed to avoid excess of contaminating leucocytes. AdnaGen kits were used following supplier recommendations (*AdnaTest EMT-1/Stem CellSelect*, AdnaGen AG, Langenhagen, Germany).

### Detection

Then, *AdnaTest EMT-1/Stem CellDetect *kit was used, according to supplier information (AdnaGen AG, Langenhagen Germany). The analysis of tumor-associated mRNA, isolated from tumor cells, was performed by RT-PCR for the following transcripts: PI3Kα, Twist1, Akt-2 and ALDH1. Thermal profiles used are those recommended by the supplier.

We added CD44 and Bmi1 as subsidiary markers of stemness. For each one, RT sample previously obtained was amplified by singleplex PCR. Sequences of primers are: CD44 forward GCCCAATGCCTTTGATGGACC, CD44 reverse GCAGGGATTCTGTCTGTGCTG and Bmi1 forward CATTGTCTTTTCCGCCCGC, Bmi1 reverseCAAAGCACACACATCAGGTGGG. The thermal profile used for CD44 PCR was as follows: after15 min denaturation at 95°C, 33 cycles of PCR were carried out by denaturation at 94°C for 30 s, annealing/extension at 59°C for 30 s, and elongation for 72°C for 30 s. Termination of the PCR reaction was subsequently carried out at 72°C for 5 min followed by storage of the sample at 10°C. The one for Bmi1 was as follows: after 15 min denaturation at 95°C, 36 cycles of PCR were carried out by denaturation at 94°C for 30 s, annealing/extension at 59.7°C for 30 s, and elongation for 72°C for 30 s. Termination of the PCR reaction was subsequently carried out at 72°C for 5 min followed by storage of the sample at 10°C. The primers generate fragments of the following sizes (257 bp CD44 -132 bp Bmi1).

Sensiscript and HotStarTaq from Qiagen GmbH (Hilden, Germany) were used for RT and amplication of cDNA templates.

### Electrophoretic analysis of PCR fragments

Visualization of PCR fragments was carried out with 2100 bioanalyser (Dna1000 LabChip Agilent technologies). Peaks were considered as positive when concentration was ≥0.15 ng/μL (according to AdnaGen indications). CD44 and Bmi1 expressions were considered positive when transcript concentration was above 0.50 ng/μl. Blood collected from 20 healthy donors was investigated to determine this cut-off value. To confirm it, larger investigation will be implemented.

### Statistical Analysis

Statistical analysis were performed using Software XLStat2011. Khi2 test was used to establish a relationship between ddCTC detection and tumor characteristics or lymphatic invasion. As the size of the sample was small we controled Khi2 test results by using the Barnet Woolf [18] and Adjusted R Squared tests.

## Results

### Patient characteristics

All patients (aged from 40 to 75 years, average 66 years) were studied at the time of newly diagnosed breast cancer and have been included after elimination of bone visceral and cerebral metastasis, whether they had or not an axillary lymphatic node invasion. Analyses were performed before any therapy. Table 1 describes clinical and pathologists' characteristics of the tumor. This table indicated the major features of each tumor and their molecular characteristics as proposed in numerous publications [19,20]. We distinguished four tumor types: luminal A (53 cases), luminal B (3 cases), overexpressed Her-2 (1 case) and triple negative carcinoma (4 cases). Values (mean ± SD) of Ki67 index were respectively for these tumor types: 11(± 7), 27 (± 3), 30, and 13 (± 3). Infiltrant lobular or infiltrant ductal carcinoma were indicated. Most of the patients had no positive axillary lymph nodes (45 upon 61); Four primary tumors expressed Her-2 (Dako, score 3 plus/Fish). Estrogen receptors (ER) were expressed in 95% of examined tumors.

**Table 1:** Primary tumour and lymph node status of the total cohort

Patient cohort characteristics		Cohort
**Number**		**61**

**Histology**	Invasive ductal carcinoma	47

	Invasive lobular carcinoma	11

	Others	3

**Molecular**	Luminal A	53

**Characteristics**	Luminal B	3

	Triple negative	4

	Her2 overexpression	1

**Size tumour**	T1	29

	T2	28

	T3 and T4	4

**Lymph node status**	N+	16

	N-	45

**Tumor grade**	I	13

	II	36

	III	9

	Unknown	3

**Hormonal**	ER+	58

**status**	PR+	46

**Her-2 status**	Her2 +	4

	Her2 -	57

### EMT-Stemness detection

**Figure 1:**
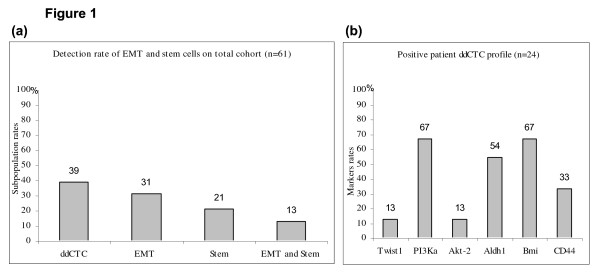
**Detection of circulating mesenchymal and cancer stem cells in early breast cancer**. **a) **Rates of ddCTC and cell subpopulations among the total cohort. **b) **Rates of EMT and stemness markers for 24 positive patients.

Among 61 patients, 24 had ddCTC expressing at least one EMT or ALDH1 markers. Thus 39% of the total cohort were positive for ddCTC (Table 2). Moreover among this population 31%, 21% and 13% showed respectively EMT cell, stem cell, both EMT and stem cell markers (Figure 1a). Rate analysis of markers for 24 positive patients is delineated in Figure 1b (Twist1 13%, PI3Kα 67%, Akt-2 13%, and ALDH1 54%). When analyses detected at least one EMT marker or ALDH1, the sample was further examined for Bmi1 and CD44. For these 2 markers, we applied a transcript cut-off value of 0.50 ng/μL (specificity of the cut-off is more than 90%, as confirmed in 20 healthy donor samples). Thus, ddCTC detected by mesenchymal and/or ALDHI markers, were 67% and 33% positive for Bmi1 and CD44 respectively. Among 45 N^- ^patients, 21 showed ddCTC and upon 16 N^+ ^patients only 3 had ddCTC.

**Table 2:** Results of ddCTC detection and statistical analysis

				Khi2	Barnet Woolf
**Patient cohort characteristics**		**negative** **ddCTC**	**positive** **ddCTC**	***P***	***P***

**Number**		**37**	**24**		

				**NS**	**NS**

**Histology**	Invasive ductal carcinoma	**28**	**19**		

	Invasive lobular carcinoma	**8**	**3**		

	Others	**1**	**2**		

				**NS**	**NS**

**Molecular**	Luminal A	**32**	**21**		

**Characteristics**	Luminal B	**2**	**1**		

	Triple negative	**2**	**2**		

	Her2 overexpression	**1**	**0**		

				**NS**	**NS**

**Size tumour**	T1	**17**	**12**		

	T2	**18**	**10**		

	T3 and T4	**2**	**2**		

				0.05*	< 0.05*

**Lymph node**	N+	**13**	**3**		

**status**					

	N-	**24**	**21**		

				**NS**	**NS**

**Tumor grade**	I	**8**	**5**		

	II	**22**	**14**		

	III	**5**	**4**		

	Unknown	**2**	**1**		

				**ND**	**ND**

**Hormonal**	ER+	**37**	**21**		

**status**	ER-	**0**	**3**		

				**NS**	**NS**

	PR+	**28**	**18**		

	PR-	**9**	**6**		

Values of *P *for Khi2 and Barnet Woolf tests are indicated for each clinicopathological characteristics of tumors

*significance level: *P *0.05, NS: *P *> 0.05 for alpha risk 5%; ND: not determined

### Statistical results

Statistical studies indicated that ddCTC detection is an independant factor of the primary tumor characteristics but not of the lymph node status (Table 2). Results of Khi 2 test were corroborated by an other reliable test: Barnet Woolf. There was a relationship between ddCTC positivity and the absence of lymph node invasion *p *= 0.050, (risk = 5%) and values of Adjusted *R^2 ^*had a significance level for = 0.05. Presence of ddCTC was predominant when patients were lymph node negative: 47% of N^- ^patients and 19% of N^+ ^patients were ddCTC positive. This correlation between lymphatic invasion and ddCTC was not previously demonstrated for patients at baseline primary breast cancer diagnosis. However we must keep in mind that lymphatic internal mammary chain has not been explored.

## Discussion

It seemed us important to develop analyses able to detect cells which are arising from EMT and spreading into the blood stream. Many arguments reported in recent literature sustained the choice of markers used in our study: PI3Kα, Akt-2, Twist1, ALDH1, Bmi1, CD44 [14-16]. In breast cancer, some patients develop metastasis many years after the apparently successful therapy of the primary cancer. The residual cancer disease is based on the notion of occult metastatic tumor cells in bone marrow. Metastasis formation from epithelial tumors progresses through dissemination of tumor cells that can follow two major ways: bloodstream or lymphatic vessels [6]. Cancer cell invasion seems proceed from EMT program [21]. The latter is associated and interacts with cellular pathways that confer new characteristics to the cells: apoptosis resistance, chemo and radio resistance. EMT can be viewed as a continuum of a progressive dedifferentiation leading to cells with stem-cell like properties. Overexpression of a few EMT regulators may be sufficient to generate the cancer stem cell phenotype [22]. A molecular link between EMT and stemness emerged with the finding that Bmi1 is a direct transcriptional target of the EMT inducer Twist1 [23]. These phenomena establish a hierarchy of cancer cell phenotypes from mesenchymal to stemness status.

Our results led to classify patients as follows. The first phenotype of ddCTC is essentially mesenchymal with markers of EMT. Among the 3 markers, the predominant one is PI3Kα, then in equal proportions Akt-2 and Twist1. The second phenotype is characterized by ALDH1 expression, a stemness marker. The third phenotype is a mixture of the two previous ones and represents 33% of positive patients.

Aktas *et al *evaluated 226 blood samples of 39 metastatic breast cancer patients during a follow-up of different therapies for the expression of the stem cell and EMT markers [16]. In 31% of detected CTC positive samples, 62% were positive for at least one of the EMT markers and 69% for ALDH1. Thus they demonstrated for the first time that a major proportion of CTC of metastatic breast cancer patients shows EMT and tumor stem cell characteristics. In another study, Kallergi *et al *identified in patients with early and metastatic breast cancer, CTC expressing Twist and vimentin. Higher incidence of these cells in metastasis disease than in early stage breast cancer supports the hypothesis that EMT is involved in metastatic potential of CTC [24]. Moreover the detection rates they reported are similar to ours. In our study, 39% of patients who were positive for EMT and ALDH1 markers had Bmi1 and CD44 positive cells in 67% and 33% respectively. The expression of Bmi1 and CD44 arose in 26% and 13% of ddCTC samples. These results support the stemness characters of these positive samples. Indeed it was shown that EMT generates cells with stem like properties [25,26] and that the stemness factor Bmi1 is regulated by Twist1 [23].

It is the first time that ddCTC presence in the blood is demonstrated with such a high rate (24/61 patients) at least when the primary breast tumor is diagnosed without metastasis. If CTC are considered as a prognostic factor, our results showed clearly that detection of all cell phenotypes should be taken into account to have an idea of the disease progression. Ongoing trials in our laboratory indicate that ddCTC are more frequently detected than CTC characterized by *AdnaTestBreastCancerSelect *and *Detect *kits (GA733-2, Muc-1 and Her-2) in primary breast cancer patients. Preliminary results, upon 400 patients showed that 8% of patients are positive for CTC according to this previous basic AdnaGen kit (data not shown). This result is not so far from those of the literature. Banys et al. found 12% of positive patients upon about 209, by using the same analytical test [17]. Braun *et al *demonstrated that incidence of bone marrow micrometastasis (disseminated tumor cells) was similar in patients with lymph node metastasis and those whithout [27]. Moreover Menard *et al *suggested that lymph node metastasis are not necessarily associated with cancer hematogenous spread [28]. Our results stress that absence of lymph node invasion is not a criteria of non dissemination. Among 45 N^- ^patients 47% showed a ddCTC positive profile and upon 16 N^+ ^patients only 19% had a positive profile. Nevertheless such a result does not weaken the negative pronostic value of lymph node invasion. These results could be explained as indicated by Chaffer and Weinberg that spread to other sites occurs mostly via the blood stream [6]. Our results are in agreement with their observations. Albeit statistical results indicated a relationship between axillary node invasion and detection of ddCTC, the significance level had a borderline value (*p *= 0.05). These preliminary statistical results have to be confirmed by a larger trial avoiding an inaccurate inference of Khi2.

Some methods for CTC analyses detect entities that by all criteria are cancer cells but which lack the ability to invade, proliferate and cause metastasis [29]. We can speculate according to these previous publications, that the CTC population is an heterogeneous one. According to these observations, our results showed dedifferentiated EMT and stemness subpopulations in primary breast cancer patients. Such a discrimination had been suggested by Mego *et al. *[30]. Indeed they demonstrated that metastatic breast cancer patients, had substantially different prognosis correlated to CTC subpopulations. A future prospective study is warranted to determine the incidence of ddCTC characterization on the prognosis. We believe that follow-up of ddCTC may facilitate the monitoring of therapeutic agents targeting these cells.

## Conclusions

Most CTC studies have been conducted in metastatic breast cancer to establish the prognostic value of this marker. Very few studies have been devoted to CTC in primary breast cancer. In a cohort of patients without metastasis, our results showed that ddCTC with EMT and stemness characteristics can be detected. Although they are tumor initiating cells, majority of them are often not detected by generally used technologies in clinical trials. The demonstration of EMT program activation, leading to invasive properties and stemness of cancer cells, seems to be a new way to characterize CTC. The relationship between ddCTC and lymph node invasion suggests that blood is a major dissemination route. The disease status should be analyzed by the quantitative measurement of EMT gene expression. It could be efficient to control new drugs targeting mesenchymal cells. Eradication of ddCTC would be the proof of therapeutic effectiveness in clinical trials.

## Abbreviations

ddCTC: Dedifferentiated circulating tumor cells; EMT: Epithelial to mesenchymal transition; CTC: Circulating tumor cell.

## Competing interests

The authors declare that they have no competing interests.

## Authors' contributions

All contributors participated equally to this study. All authors read and approved the final manuscript.

## Pre-publication history

The pre-publication history for this paper can be accessed here:

http://www.biomedcentral.com/1471-2407/12/114/prepub

## Aknowledgements

Our work was financially supported by URCAM- Limousin (Union Régionale des Caisses d'Assurance Maladie). Our special thanks to Pr Fernand Boutros-Toni (departement of Epidemiology and Statistics- Medicine Faculty of Limoges) for statistical analysis.
